# Dopaminergic modulation of propofol-induced activation in VLPO neurons: the role of D1 receptors in sleep-promoting neural circuits

**DOI:** 10.3389/fnins.2024.1485873

**Published:** 2025-01-08

**Authors:** Kun Qian, Yu Zhang, Yang Liu, Sisi Wu, Zikun Duan, Jianhao Liao, Wei Luo, Mo Zhou, Xuejiao Dou, Xingkui Liu, Tian Yu

**Affiliations:** ^1^Department of Anesthesiology, Affiliated Hospital of Zunyi Medical University, Zunyi, China; ^2^The Key Laboratory of Anesthesia and Organ Protection, The Key Laboratory of Brain Science, Zunyi Medical University, Zunyi, China; ^3^College of Anesthesiology, Zunyi Medical University, Zunyi, China

**Keywords:** ventrolateral preoptic nucleus, propofol, spontaneous excitatory postsynaptic currents, spontaneous inhibitory postsynaptic currents, D1 dopaminergic receptors

## Abstract

**Background:**

The ventrolateral preoptic nucleus (VLPO) is a crucial regulator of sleep, and its neurons are implicated in both sleep-wake regulation and anesthesia-induced loss of consciousness. Propofol (PRO), a widely used intravenous anesthetic, modulates the activity of VLPO neurons, but the underlying mechanisms, particularly the role of dopaminergic receptors, remain unclear.

**Objective:**

This study aimed to investigate the effects of PRO on NA (−) neurons in the VLPO and to determine the involvement of D1 and D2 dopaminergic receptors in mediating these effects.

**Methods:**

Using *in vitro* patch-clamp techniques, we identified and characterized NA (−) and NA (+) neurons in the VLPO based on their morphological, pharmacological, and electrophysiological properties. We assessed the effects of PRO on spontaneous excitatory postsynaptic currents (sEPSCs) and inhibitory postsynaptic currents (sIPSCs) in NA (−) neurons, both in the presence and absence of dopaminergic receptor modulators.

**Results:**

PRO significantly increased the firing frequency of NA (−) neurons while decreasing the firing frequency of NA (+) neurons. This activation of NA (−) neurons was mediated through GABA_A receptors, as evidenced by the increased frequency of sEPSCs and altered sIPSCs dynamics. Dopamine (DA) attenuated the PRO-induced increase in sEPSCs frequency and suppression of sIPSCs frequency in NA (−) neurons via D1 receptors, but not D2 receptors. Blocking D1 receptors with SCH23390 reversed the effects of DA on PRO-induced changes, while D2 receptor antagonist sulpiride had minimal impact.

**Conclusion:**

Our findings demonstrate that PRO excites sleep-promoting NA (−) neurons in the VLPO, primarily through GABA_A receptors, with dopaminergic modulation occurring via D1 receptors. These results provide new insights into the neural mechanisms underlying general anesthesia and highlight the potential role of dopaminergic signaling in modulating anesthetic effects on sleep-related neural circuits.

## Graphical abstract

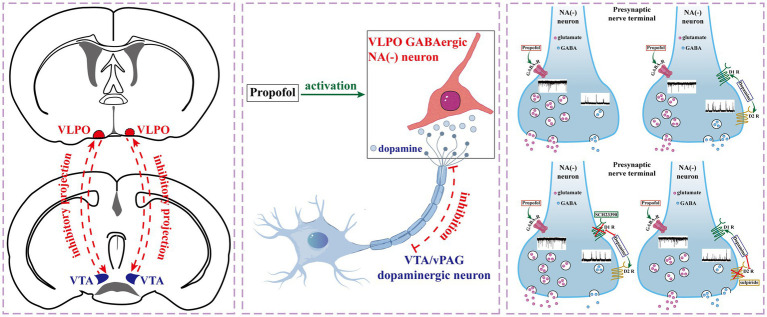

## 1 Introduction

General anesthesia has been a cornerstone of modern clinical practice for over 180 years, enabling reversible loss of consciousness (LOC) in billions of patients annually (Mashour, 2024). Despite its widespread use, the neural and cellular mechanisms underlying general anesthesia remain incompletely understood (Gao et al., 2024; Pavel et al., 2020). Although LOC induced by anesthetics and natural sleep are distinct states, they share significant similarities, leading to the hypothesis that both may involve overlapping neural circuits responsible for inducing reversible unconsciousness (Bao et al., 2023; Moody et al., 2021; Eikermann et al., 2020; Yang et al., 2022; Franks and Wisden, 2021).

The ventrolateral preoptic nucleus (VLPO) is recognized as a critical regulator of sleep (Arrigoni and Fuller, 2022; Liang et al., 2021; Kostin et al., 2023; Huo et al., 2024), composed primarily of three neuronal populations (Chung et al., 2017; Prokofeva et al., 2023; Dubourget et al., 2016): GABAergic, glutamatergic, and galaninergic neurons. Among these, GABAergic neurons are particularly noted for their role in promoting sleep (Yang et al., 2022; Kostin et al., 2023; Oishi et al., 2023). There is considerable evidence suggesting that specific VLPO neurons, particularly those inhibited by wake-promoting neurotransmitters such as noradrenaline (NA) (Fan et al., 2023; Liu et al., 2017; Weber and Dan, 2016), acetylcholine (ACh), and serotonin (5-HT) (Weber and Dan, 2016), are activated by intravenous anesthetic propofol (PRO) and inhaled anesthetic isoflurane (Liu et al., 2017; Luo et al., 2023).

Previous studies have demonstrated that hypnotic doses of PRO increase c-Fos expression in the VLPO (Yue et al., 2021), indicating heightened neuronal activity, similar to that observed with isoflurane (Han et al., 2014). This activation is thought to result from the inhibition of GABAergic transmission to VLPO neurons, leading to their disinhibition and subsequent induction of LOC (Yuan et al., 2017). Additionally, recent studies have shown that activation of VLPO GABAergic or glutamatergic neurons is essential for facilitating PRO-induced sedation (Yuan et al., 2017; Zhang et al., 2015), although the effects of these activations on isoflurane anesthesia remain controversial (Vanini et al., 2020).

The VLPO is interconnected with several wake-promoting nuclei, including the dopaminergic ventral tegmental area (VTA) (Bao et al., 2023; Yang et al., 2022) and ventral periaqueductal gray (vPAG) (Weber and Dan, 2016). These dopaminergic projections play a key role in modulating VLPO activity, primarily through D1 and D2 receptors expressed on sleep-promoting GABAergic neurons (Weber and Dan, 2016; Luo et al., 2023; Liu et al., 2020; Vincent et al., 2024). Studies such as those by Cameron et al. (1995), Gordon-Fennell et al. (2019), and Cornil et al. (2002) provide anatomical evidence of projections from the VTA and vPAG to the preoptic area, underscoring the functional connectivity of these circuits. Functional studies further suggest that VLPO-mediated inhibition of arousal-promoting nuclei, coupled with dopaminergic input, contributes to sleep-wake regulation and anesthetic mechanisms (Gordon-Fennell et al., 2020; Miller and Lonstein, 2009). The activation of D1 receptors in the VTA, for instance, has been shown to facilitate emergence from anesthesia induced by agents such as sevoflurane, isoflurane, and fentanyl (Vincent et al., 2024). These findings highlight the importance of dopaminergic signaling in regulating the neural circuits underlying LOC and recovery.

However, the specific role of DA receptors on VLPO neurons in modulating the effects of PRO remains unclear. This study aims to investigate how DA receptors, particularly D1 receptors, influence the effects of PRO on VLPO neurons *in vitro*. We hypothesize that DA modulates the excitatory and inhibitory effects of PRO on different neuron types within the VLPO, providing new insights into the neural mechanisms underlying general anesthesia.

This study aims to investigate how DA receptors, particularly D1 receptors, influence the effects of PRO on VLPO neurons *in vitro*. We hypothesize that DA modulates the excitatory and inhibitory effects of PRO on different neuron types within the VLPO, providing new insights into the neural mechanisms underlying general anesthesia.

## 2 Methods

### 2.1 Animals

All experimental procedures adhered to the guidelines of the Guide for the Care and Use of Laboratory Animals and were approved by the Animal Care and Use Committees of Zunyi Medical University (ZMC2014-0901). Efforts were made to minimize animal suffering and reduce the number of animals used in the study. One hundred and twenty SPF-grade neonatal male Sprague–Dawley (SD) rats (weighing 10–25 g, aged 7–14 days) were obtained from the Animal Center of the Third Military Medical University [license number: SCXK (Yu) 2012-0005; Chongqing, China]. The rats were housed in polyvinyl chloride cages within an SPF-grade animal facility at the Guizhou Key Laboratory of Anesthesia and Organ Protection, equipped with an independent ventilation system maintaining a constant temperature of 24 ± 2°C and humidity of 60 ± 2%. The animals were kept on a 12-h light/dark cycle (lights on at 7:00 am and off at 7:00 pm) with free access to food and water.

### 2.2 Chemicals and application

PRO and isoflurane were obtained from AstraZeneca (London, United Kingdom) and RWD Life Science (Shenzhen, China), respectively. Noradrenaline (NA), GABAzine, DL-2-amino-5-phosphonovaleric acid (AP-5), 6,7-dinitroquinoxaline-2,3-dione (DNQX), strychnine, DA, SCH23390, sulpiride, cesium methanesulfonate, cesium fluoride (CsF), potassium gluconate, Mg-ATP, adenosine 5′-triphosphate (GTP), and hydroxyethyl piperazine ethanesulfonic acid (HEPES) were purchased from Sigma. All drugs and solutions were applied to the neurons using a perfusion system driven by a peristaltic pump (BPS-4, ALA, United States).

### 2.3 Brain slice preparation

As previously described (Liu et al., 2017), male SD rats (aged 7–14 days) were decapitated under isoflurane anesthesia, and the brains were rapidly transferred to ice-cold (0–4°C) modified artificial cerebrospinal fluid (ACSF) saturated with 95% O_2_ and 5% CO_2_. The ACSF solution contained (in mM): 126 NaCl, 3 KCl, 2.4 CaCl_2_, 1.2 MgCl_2_, 11 glucose, 1.4 NaH_2_PO_4_, and 25 NaHCO_3_ (osmolality adjusted to 310 mOsm with sucrose, pH adjusted to 7.2 with CsOH). Coronal hypothalamic slices containing the VLPO (250–300 μm thick) were prepared using a Thermo HM 650 V slicer (HM650V, Thermo, United States). The slices were incubated at 32°C for 30 min and then allowed to recover at room temperature (22–25°C) in ACSF aerated with 95% O_2_ and 5% CO_2_ for at least 30 min before electrophysiological recordings.

### 2.4 Patch-clamp electrophysiological recordings in brain slices

Following recovery, brain slices containing the VLPO were transferred to a recording chamber mounted on an auto-balance vibration isolation table (VH, Newport, United States) and held in place with a flat U-shaped wire. The VLPO region was visually identified under a fluorescence confocal microscope (BX51W1-IR7, Olympus, Japan) using a low-power lens and based on the rat brain stereotaxic atlas. Healthy neurons were identified by their refractive surface, full cytoplasm, and clearly visible axons and dendrites. Patch micropipettes (loose-patch: 1 MΩ; whole-cell: 5–7 MΩ) were pulled from borosilicate glass capillaries using a micropipette puller (P-97, Sutter, United States). Neurons were approached under visual guidance using a three-axis microelectrode manipulator system (PCS-5400, Burleigh, United States). Signals were amplified by a diaphragm clamp amplifier (EPC10, HEKA, Germany), low-pass filtered at 3 kHz, and sampled at a frequency of 20 kHz.

### 2.5 Loose-patch cell-attached identification of VLPO neurons

During recordings, brain slices were submerged in ACSF continuously bubbled with 95% O_2_ and 5% CO_2_ (2 mL/min) at 22–25°C. Previous studies have shown that about two-thirds of VLPO sleep-promoting neurons with low-threshold spikes (LTS) are multipolar or triangular and are inhibited by NA (Fan et al., 2023; Weber and Dan, 2016). In contrast, the remaining neurons, which are fusiform or bipolar, are excited by NA (Fan et al., 2023). We used microscopy to identify these two primary types of neurons within the VLPO based on their location, cellular morphology, and electrophysiological properties. For spike recordings, patch electrodes (1 MΩ) were filled with a solution containing (in mM): 140 potassium gluconate, 5 KCl, 2 MgCl_2_, 10 HEPES, 2 Mg-ATP, and 0.2 GTP (310 mOsm, pH 7.2). The majority of neurons (69.39%, 34 out of 49) in our study were classified as NA (−) neurons, displaying triangular or multipolar morphology, with spikes inhibited by NA (100 μM) and reversible after washout. The remaining neurons (30.61%, 15 out of 49) were classified as NA (+) neurons, characterized by bipolar or fusiform morphology, with spikes excited by NA (100 μM). It is worth emphasizing that further studies will concern only the category of NA (−) neurons.

### 2.6 Whole-cell patch-clamp recordings for sleep-promoting NA (−) neurons

#### 2.6.1 Experimental groups

One hundred and twenty rats were randomly divided into nine groups: control, PRO, wash, DA, PRO + DA, SCH23390 + DA, PRO + SCH23390 + DA, sulpiride + DA, and PRO + sulpiride + DA. In the PRO group, neuronal firing, sEPSCs, and sIPSCs of NA (−) neurons were recorded before and after a 5-min perfusion of PRO (10 μM). In the DA group, changes in sEPSCs and sIPSCs were recorded before and after a 5-min perfusion of PRO (10 μM) under continuous perfusion of DA (100 μM). In the SCH23390 and sulpiride groups, changes in sEPSCs and sIPSCs were recorded after adding DA (100 μM) and then during perfusion of PRO (10 μM) + DA (100 μM) under continuous perfusion with the D1 receptor antagonist SCH23390 (10 μM) or the D2 receptor antagonist sulpiride (10 μM).

#### 2.6.2 Whole-cell patch-clamp recordings

For whole-cell patch-clamp recordings, slight negative pressure was applied to the microelectrode upon contact with the cell membrane of NA (−) neurons, forming a high-resistance seal (greater than 1 GΩ). Further suction was applied to rupture the membrane and establish the whole-cell recording mode, ensuring that membrane resistance remained greater than or equal to 150 MΩ. Series resistance compensation was set to not exceed 30%, and cells with higher compensation were discarded.

Action potentials of VLPO neurons were recorded in the whole-cell current-clamp mode. For sEPSC recordings, potassium gluconate in the intracellular solution was replaced with CsF (135 mM), and sEPSCs were recorded at a holding potential of −60 mV. For sIPSC recordings, cesium methanesulfonate was used instead of potassium gluconate in the pipette solution, and sIPSCs were recorded at 0 mV. The bath ACSF solution contained 50 μM AP5, 20 μM DNQX, and 1 μM strychnine. Data were analyzed using Clampfit 10.7 software (Molecular Devices, United States).

### 2.7 Statistical analysis

Electrical signals were measured and analyzed using Clampfit 10.7 software and a HEKA EPC10 amplifier. Data were plotted using GraphPad Prism 9.3. All experimental data are presented as means ± standard deviation, with “*n*” referring to the number of neurons tested. Changes in results before and after the administration of PRO or dopaminergic receptor antagonists were compared using paired *t*-tests or one-way analysis of variance (ANOVA). The Student–Newman–Keuls *post hoc* test was employed for ANOVA. A *p*-value of <0.05 was considered statistically significant.

## 3 Results

### 3.1 Propofol (PRO) excites NA (−) neurons and inhibits NA (+) neurons in the VLPO

**Figure 1:**
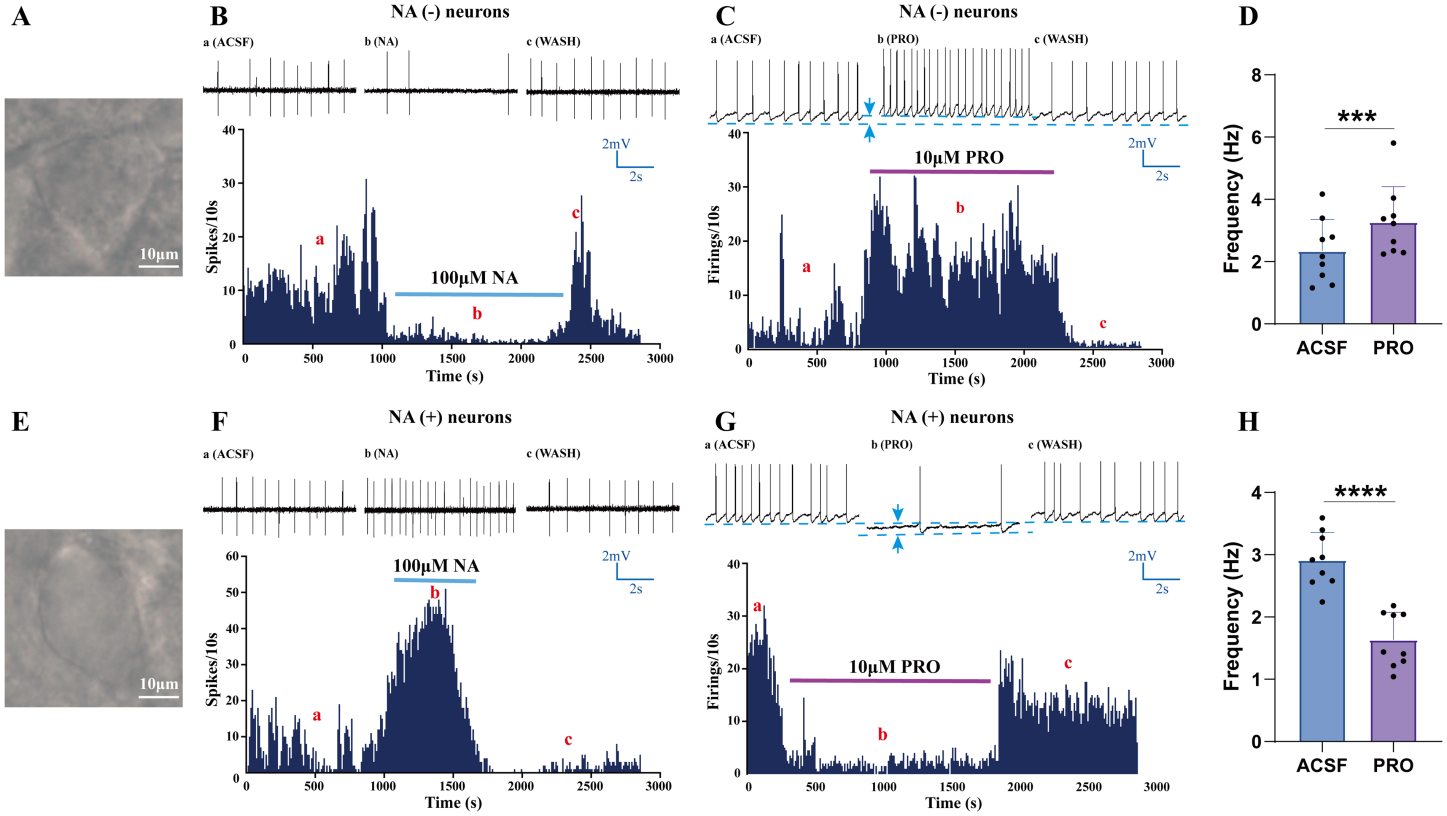
Identification and characterization of NA (−) and NA (+) neurons in the VLPO. **(A)** Bright-field image of a typical triangular-shaped NA (−) neuron in the VLPO. **(B)** Loose-patch cell-attached recordings showing that bath-applied noradrenaline (NA, 100 μM) inhibited the firing of NA (−) neurons. **(C)** Whole-cell patch-clamp recordings in current-clamp mode demonstrated that propofol (PRO, 10 μM) increased the spontaneous firing frequency of NA (−) neurons. **(D)** Statistical analysis of the effect of PRO on the firing frequency of NA (−) neurons (*n* = 9, ^***^p < 0.001). **(E)** Bright-field image of a typical fusiform-shaped NA (+) neuron in the VLPO. **(F)** Loose-patch cell-attached recordings showing that NA (100 μM) excited the firing of NA (+) neurons. **(G)** Whole-cell patch-clamp recordings in current-clamp mode demonstrated that PRO (10 μM) decreased the spontaneous firing frequency of NA (+) neurons. **(H)** Statistical analysis of the effect of PRO on the firing frequency of NA (+) neurons (*n* = 9, ^****^p < 0.0001). Data are presented as mean ± standard deviation and analyzed using paired *t*-tests. ^***^p < 0.001 and ^****^p < 0.0001.

Consistent with the electrophysiological characteristics reported in previous studies (Chung et al., 2017; Fan et al., 2023), we first identified and classified VLPO neurons based on their response to noradrenaline (NA) in acute hypothalamic slices from rats (Figures 1B,F). Triangular-shaped neurons in the VLPO were categorized as NA (−) neurons because their ongoing discharges were inhibited by NA (100 μM) in cell-attached recordings (Figures 1A,B). In contrast, the fusiform-shaped neurons, which exhibited increased spike activity in response to NA, were classified as NA (+) neurons (Figures 1E,F).

To assess the effects of the general anesthetic PRO on these distinct VLPO neuronal populations, we employed whole-cell patch-clamp recordings in current-clamp mode (Figures 1C,G). Our results showed that PRO (10 μM) significantly increased the action potential firing frequency of NA (−) neurons compared to their baseline activity (ACSF: 2.35 ± 1.01 Hz; PRO: 3.27 ± 1.14 Hz; *n* = 9, *p* = 0.0003; Figures 1C,D). Additionally, PRO depolarized the membrane potential of NA (−) neurons (ACSF: −52.31 ± 4.22 mV; PRO: −48.5 ± 4.1 mV; *n* = 9; Figure 1C). These effects were partially reversible upon washout with ACSF.

Conversely, PRO (10 μM) significantly decreased the firing frequency of NA (+) neurons (ACSF: 2.91 ± 0.44 Hz; PRO: 1.64 ± 0.44 Hz; *n* = 9, *p* < 0.0001; Figures 1G,H) and induced hyperpolarization of their membrane potential (ACSF: −45.31 ± 5.13 mV; PRO: −49.50 ± 5.30 mV; *n* = 9; Figure 1G). These findings suggest that PRO enhances the excitability of sleep-promoting NA (−) neurons while concurrently inhibiting wake-promoting NA (+) neurons in the VLPO.

### 3.2 PRO increases glutamatergic sEPSCs frequency and modulates GABAergic sIPSCs in NA (−) neurons via GABA_A receptors

**Figure 2:**
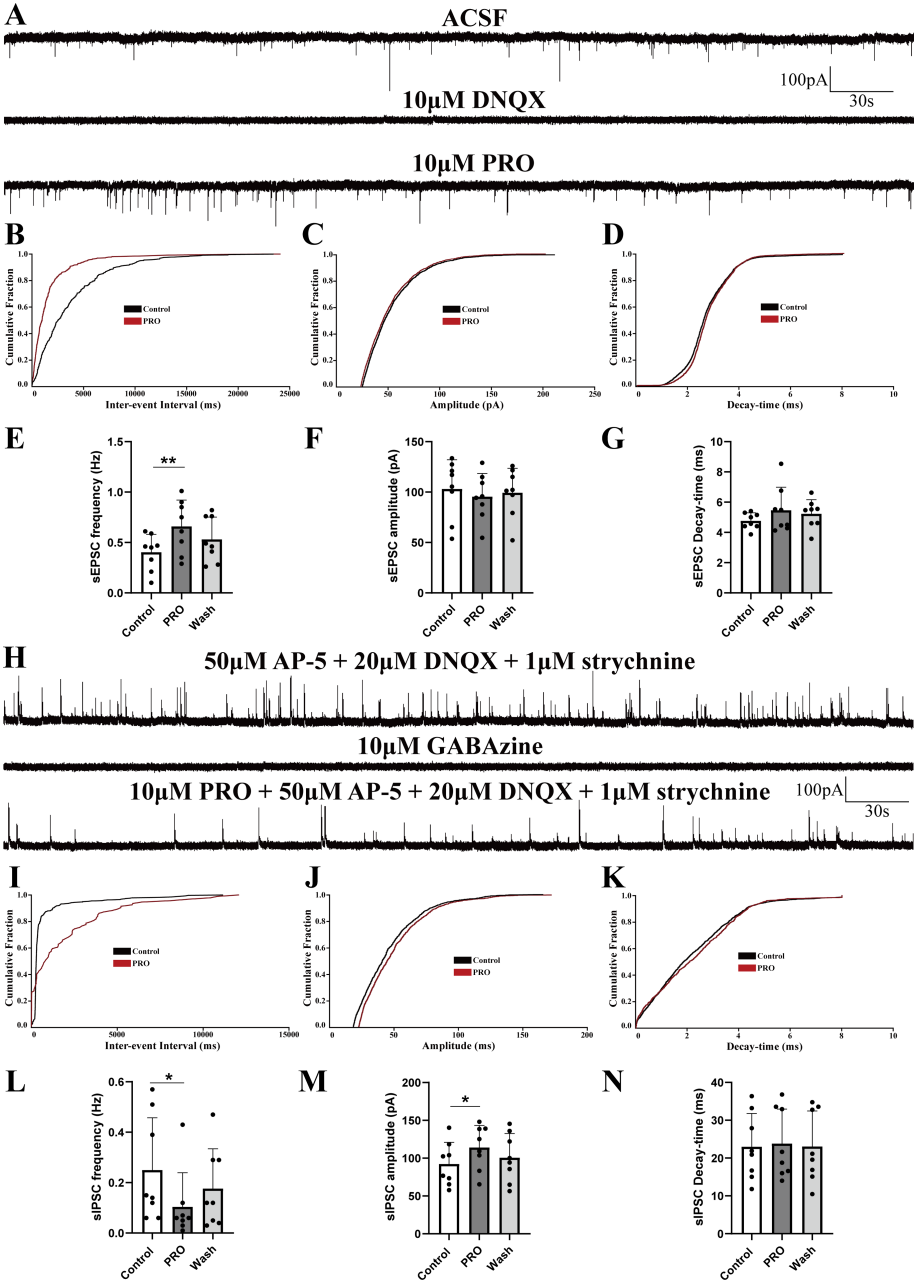
Propofol (PRO) modulates sEPSCs and sIPSCs in NA (−) neurons of the VLPO. **(A)** Representative traces showing AMPA receptor-mediated spontaneous excitatory postsynaptic currents (sEPSCs) recorded in NA (−) neurons. **(B)** Cumulative probability plot showing the inter-event intervals of sEPSCs during control (ACSF) and PRO (10 μM) conditions. **(C)** Cumulative probability plot of sEPSCs amplitude in control (ACSF) and PRO conditions. **(D)** Cumulative probability plot of sEPSCs decay-time in control (ACSF) and PRO conditions. **(E)** Statistical analysis of sEPSCs frequency before and after PRO application (*n* = 8, ^**^p < 0.01). **(F)** Statistical analysis of sEPSCs amplitude before and after PRO application (*n* = 8, *p* > 0.05). **(G)** Statistical analysis of sEPSCs decay-time before and after PRO application (*n* = 8, *p* > 0.05). **(H)** Representative traces showing GABA_A receptor-mediated spontaneous inhibitory postsynaptic currents (sIPSCs) recorded in NA (−) neurons. **(I)** Cumulative probability plot showing the inter-event intervals of sIPSCs during control (ACSF) and PRO (10 μM) conditions. **(J)** Cumulative probability plot of sIPSCs amplitude in control (ACSF) and PRO conditions. **(K)** Cumulative probability plot of sIPSCs decay-time in control (ACSF) and PRO conditions. **(L)** Statistical analysis of sIPSCs frequency before and after PRO application (*n* = 8, ^*^p < 0.05). **(M)** Statistical analysis of sIPSCs amplitude before and after PRO application (*n* = 8, ^*^p < 0.05). **(N)** Statistical analysis of sIPSCs decay-time before and after PRO application (*n* = 8, *p* > 0.05). Data are presented as mean ± standard deviation and analyzed using one-way ANOVA. ^*^p < 0.05 and ^**^p < 0.01.

In subsequent experiments, we focused on NA (−) neurons within the VLPO, which are known to be crucial for sleep promotion. To investigate how PRO modulates synaptic transmission and contributes to the excitatory effects on NA (−) neurons, we conducted whole-cell recordings in voltage-clamp mode at a holding potential of −60 mV. As expected, the AMPA receptor-mediated spontaneous excitatory postsynaptic currents (sEPSCs) in NA (−) neurons were completely abolished by superfusion with DNQX (10 μM), an AMPA receptor antagonist, confirming that these sEPSCs were glutamatergic in nature (Figure 2A).

Our findings revealed that PRO (10 μM) significantly increased the frequency of sEPSCs in NA (−) neurons from 0.40 ± 0.18 Hz to 0.66 ± 0.26 Hz (*n* = 8, *p* = 0.003; Figure 2E). However, PRO did not significantly alter the amplitude (control: 103.01 ± 29.10 pA vs. PRO: 95.44 ± 23.06 pA; *n* = 8, *p* = 0.293; Figure 2F) or decay-time (control: 4.76 ± 0.54 ms vs. PRO: 5.46 ± 1.53 ms; *n* = 8, *p* = 0.287; Figure 2G). These effects were partially reversed following washout with ACSF (Figures 2E–G). The cumulative probability plots of the inter-event interval for sEPSCs during PRO application exhibited a significant leftward shift compared to the control, indicating an increase in sEPSCs frequency without significant changes in amplitude or decay-time (Figures 2B–D). These results suggest that PRO enhances the frequency of glutamatergic sEPSCs in NA (−) neurons in the VLPO.

To determine the role of GABA_A receptors in this modulation, we pharmacologically blocked these receptors by adding GABAzine (10 μM) to the ACSF. Under these conditions, the PRO-induced increase in sEPSCs frequency in NA (−) neurons was abolished, indicating that PRO exerts its effects through GABA_A receptor activity.

We further examined sIPSCs under similar conditions by blocking NMDA, glutamate, and glycine receptors with AP-5 (50 μM), DNQX (20 μM), and strychnine (1 μM), simultaneously, in *ex vivo* whole-cell voltage-clamp recordings at a holding potential of 0 mV (Figure 2H). Our results demonstrated that sIPSCs in NA (−) neurons were mediated by GABA_A receptors, as evidenced by their near-complete blockade with GABAzine (10 μM, Figure 2H). PRO (10 μM) significantly reduced the frequency of sIPSCs (control: 0.25 ± 0.21 Hz vs. PRO: 0.10 ± 0.14 Hz; *n* = 8, *p* = 0.037; Figure 2L) while increasing their amplitude (control: 92.28 ± 28.59 pA vs. PRO: 114.07 ± 29.16 pA; *n* = 8, *p* = 0.038; Figure 2M). The decay-time of sIPSCs was not significantly affected by PRO (control: 22.99 ± 8.79 ms vs. PRO: 23.80 ± 9.16 ms; *n* = 8, *p* = 0.595; Figure 2N). The cumulative distributions for the frequency and amplitude of sIPSCs during PRO application showed significant shifts compared to the control, suggesting that PRO decreases the frequency and increases the amplitude of sIPSCs (Figures 2I–K). These results indicate that PRO excites sleep-active NA (−) neurons in the VLPO by modulating both glutamatergic and GABAergic synaptic transmission.

### 3.3 Dopamine (DA) modulates PRO-induced changes in sEPSCs and sIPSCs in NA (−) neurons

The VLPO sends inhibitory projections to the VTA, a key arousal-regulating structure primarily composed of dopaminergic, GABAergic, and glutamatergic neurons (Oishi et al., 2023; Hou et al., 2024; Yu et al., 2022). Building on our earlier findings, we next examined how propofol (PRO, 10 μM) affects AMPA receptor-mediated sEPSCs and GABA_A receptor-mediated sIPSCs in triangular-shaped NA (−) neurons, specifically in the presence of dopamine (DA, 100 μM).

**Figure 3:**
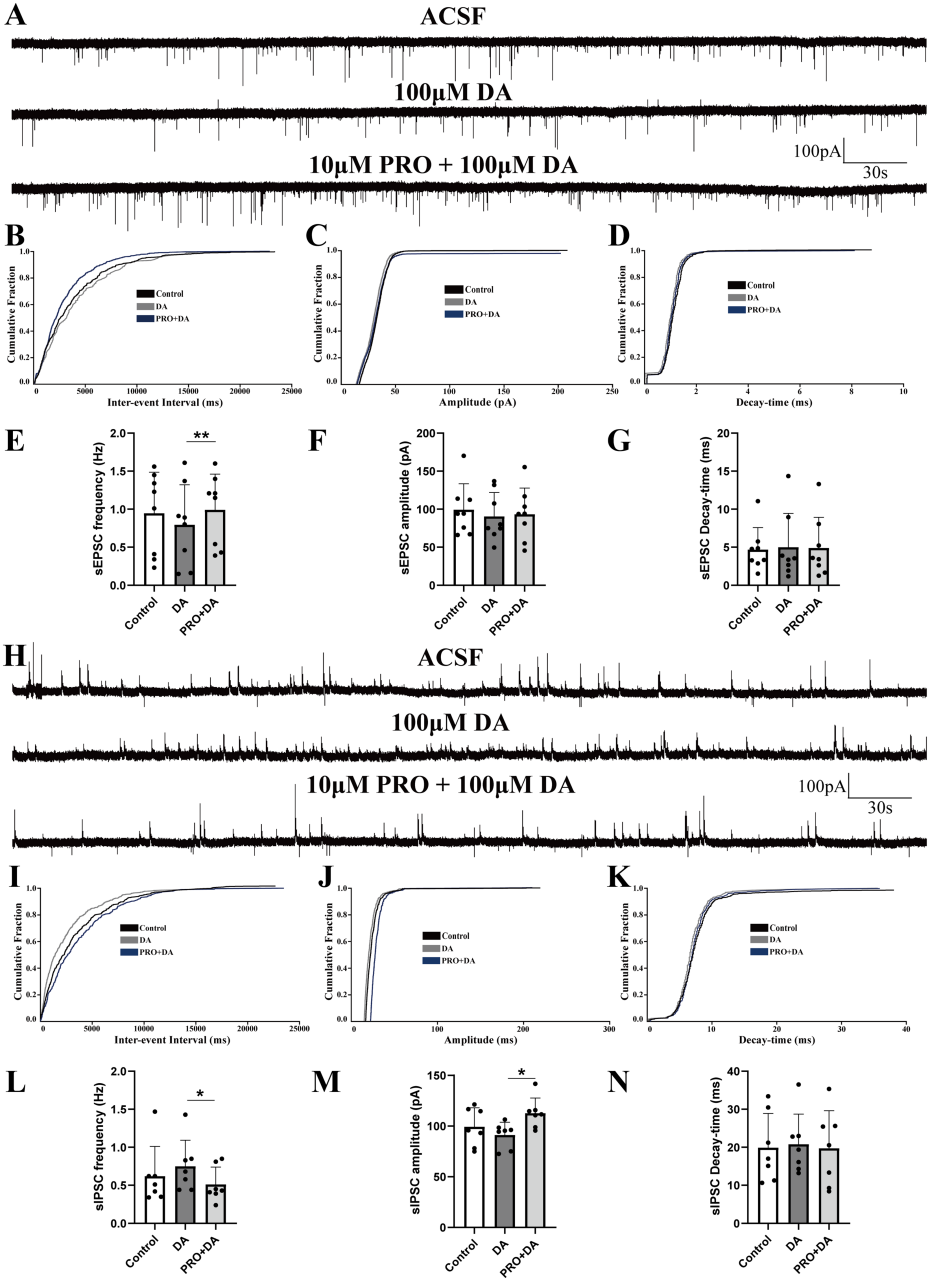
Dopamine (DA) modulates the effects of PRO on sEPSCs and sIPSCs in NA (−) neurons of the VLPO. **(A)** Representative traces showing sEPSCs recorded in NA (−) neurons during DA (100 μM) and PRO (10 μM) + DA application. **(B)** Cumulative probability plot showing the inter-event intervals of sEPSCs during control (ACSF), DA and PRO + DA conditions. **(C)** Cumulative probability plot of sEPSCs amplitude during control (ACSF), DA and PRO + DA conditions. **(D)** Cumulative probability plot of sEPSCs decay-time during Control (ACSF), DA and PRO + DA conditions. **(E)** Statistical analysis of sEPSCs frequency before and after PRO + DA application (*n* = 8, ^**^p < 0.01). **(F)** Statistical analysis of sEPSCs amplitude before and after PRO + DA application (*n* = 8, *p* > 0.05). **(G)** Statistical analysis of sEPSCs decay-time before and after PRO + DA application (*n* = 8, *p* > 0.05). **(H)** Representative traces showing sIPSCs recorded in NA (−) neurons during DA (100 μM) and PRO (10 μM) + DA application. **(I)** Cumulative probability plot showing the inter-event intervals of sIPSCs during control (ACSF), DA and PRO + DA conditions. **(J)** Cumulative probability plot of sIPSCs amplitude during control (ACSF), DA and PRO + DA conditions. **(K)** Cumulative probability plot of sIPSCs decay-time during control (ACSF), DA and PRO + DA conditions. **(L)** Statistical analysis of sIPSCs frequency before and after PRO + DA application (*n* = 7, ^*^p < 0.05). **(M)** Statistical analysis of sIPSCs amplitude before and after PRO + DA application (*n* = 7, ^*^p < 0.05). **(N)** Statistical analysis of sIPSCs decay-time before and after PRO + DA application (*n* = 7, *p* > 0.05). Data are presented as mean ± standard deviation and analyzed using one-way ANOVA. ^*^p < 0.05 and ^**^p < 0.01.

Superfusion with both PRO and DA significantly increased the frequency of sEPSCs compared to DA alone (DA: 0.79 ± 0.53 Hz vs. PRO + DA: 0.99 ± 0.47 Hz; *n* = 8, *p* = 0.007; Figures 3A,E), with no significant changes in amplitude (DA: 90.37 ± 31.56 pA vs. PRO + DA: 93.21 ± 34.62 pA; *n* = 8, *p* = 0.760; Figure 3F) or decay-time (DA: 4.98 ± 4.45 ms vs. PRO + DA: 4.89 ± 4.03 ms; *n* = 8, *p* = 0.733; Figure 3G). The cumulative distribution plot of sEPSCs frequency showed a significant leftward shift after the addition of PRO in the presence of DA, indicating an increase in sEPSCs frequency, while the amplitude and decay-time remained unchanged (Figures 3B–D).

In contrast, PRO (10 μM) in the presence of DA (100 μM) significantly reduced the frequency of sIPSCs in NA (−) neurons (DA: 0.75 ± 0.34 Hz vs. PRO + DA: 0.51 ± 0.23 Hz; *n* = 7, *p* = 0.039; Figures 3H,L), while significantly increasing the amplitude (DA: 91.25 ± 12.57 pA vs. PRO + DA: 112.68 ± 14.98 pA; *n* = 7, *p* = 0.019; Figure 3M). There were no significant changes in the decay-time of sIPSCs (DA: 20.77 ± 7.95 ms vs. PRO + DA: 19.72 ± 9.92 ms; *n* = 7, *p* = 0.654; Figure 3N). The cumulative distribution plots of sIPSCs frequency and amplitude showed a significant rightward shift with PRO in the presence of DA, indicating a reduction in sIPSCs frequency and an increase in amplitude, without affecting the decay-time (Figures 3I–K).

These findings suggest that even in the presence of DA, PRO continues to excite sleep-active NA (−) neurons in the VLPO by modulating both excitatory and inhibitory synaptic inputs.

### 3.4 Effects of SCH23390 and DA on PRO-induced changes in sEPSCs and sIPSCs in NA (−) neurons

Given that D1 and D2 receptors are the primary subtypes of dopaminergic receptors expressed on VLPO neurons, we employed dopaminergic receptor antagonists to investigate how these receptors influence the effects of PRO on sEPSCs and sIPSCs in NA (−) neurons during continuous DA application. Brain slices were continuously perfused with ACSF containing the D1 receptor antagonist SCH23390 (10 μΜ) along with DA (100 μΜ). We then compared the effects of PRO (10 μΜ) on sEPSCs and sIPSCs in NA (−) neurons across different experimental groups.

**Figure 4:**
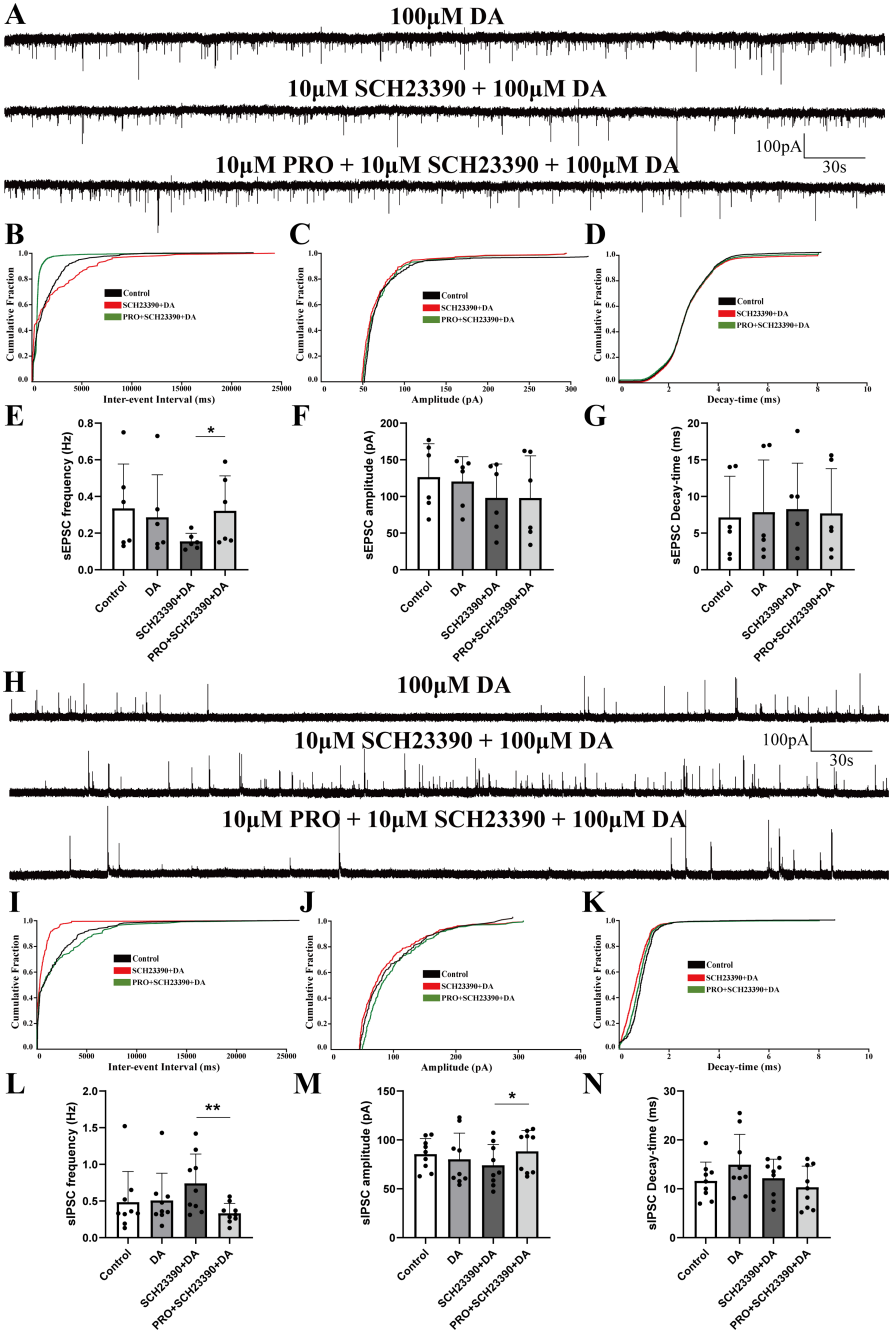
Effects of D1 receptor antagonist SCH23390 on PRO-induced changes in sEPSCs and sIPSCs in NA (−) neurons. **(A)** Representative traces showing sEPSCs recorded in NA (−) neurons during SCH23390 (10 μM) + DA (100 μM) and PRO (10 μM) + SCH23390 + DA application. **(B)** Cumulative probability plot showing the inter-event intervals of sEPSCs during control (ACSF), SCH23390 + DA and PRO + SCH23390 + DA conditions. **(C)** Cumulative probability plot of sEPSCs amplitude during control (ACSF), SCH23390 + DA and PRO + SCH23390 + DA conditions. **(D)** Cumulative probability plot of sEPSCs decay-time during control (ACSF), SCH23390 + DA and PRO + SCH23390 + DA conditions. **(E)** Statistical analysis of sEPSCs frequency before and after PRO + SCH23390 + DA application (*n* = 6, ^*^p < 0.05). **(F)** Statistical analysis of sEPSCs amplitude before and after PRO + SCH23390 + DA application (*n* = 6, *p* > 0.05). **(G)** Statistical analysis of sEPSCs decay-time before and after PRO + SCH23390 + DA application (*n* = 6, *p* > 0.05). **(H)** Representative traces showing sIPSCs recorded in NA (−) neurons during SCH23390 (10 μM) + DA (100 μM) and PRO (10 μM) + SCH23390 + DA application. **(I)** Cumulative probability plot showing the inter-event intervals of sIPSCs during control (ACSF), SCH23390 + DA and PRO + SCH23390 + DA conditions. **(J)** Cumulative probability plot of sIPSCs amplitude during control (ACSF), SCH23390 + DA and PRO + SCH23390 + DA conditions. **(K)** Cumulative probability plot of sIPSCs decay-time during control (ACSF), SCH23390 + DA and PRO + SCH23390 + DA conditions. **(L)** Statistical analysis of sIPSCs frequency before and after PRO + SCH23390 + DA application (*n* = 9, ^**^p < 0.01). **(M)** Statistical analysis of sIPSCs amplitude before and after PRO + SCH23390 + DA application (*n* = 9, ^*^p < 0.05). **(N)** Statistical analysis of sIPSCs decay-time before and after PRO + SCH23390 + DA application (*n* = 9, *p* > 0.05). Data are presented as mean ± standard deviation and analyzed using one-way ANOVA. ^*^p < 0.05 and ^**^p < 0.01.

In the presence of SCH23390 and DA, PRO significantly increased the frequency of sEPSCs compared to the SCH23390 + DA condition alone (SCH23390 + DA: 0.16 ± 0.044 Hz vs. PRO + SCH23390 + DA: 0.32 ± 0.19 Hz; *n* = 6, *p* = 0.046; Figures 4A,E). However, no significant differences were observed in the amplitude (SCH23390 + DA: 98.16 ± 46.1 pA vs. PRO + SCH23390 + DA: 97.97 ± 57.48 pA; *n* = 6, *p* = 0.994; Figure 4F) or decay-time (SCH23390 + DA: 8.27 ± 6.28 ms vs. PRO + SCH23390 + DA: 7.69 ± 6.09 ms; *n* = 6, *p* = 0.69; Figure 4G). Similarly, the cumulative probability plot of sEPSCs frequency showed a leftward shift after PRO application in the presence of SCH23390 and DA, indicating an increase in sEPSCs frequency without significant changes in amplitude or decay-time (Figures 4B–D).

As shown in Figure 4H, the frequency of sIPSCs was significantly reduced in the PRO + SCH23390 + DA group compared to the SCH23390 + DA group alone (SCH23390 + DA: 0.74 ± 0.04 Hz vs. PRO + SCH23390 + DA: 0.33 ± 0.13 Hz; *n* = 9, *p* = 0.004; Figures 4H,L). Additionally, the amplitude of sIPSCs increased significantly (SCH23390 + DA: 74.10 ± 21.24 pA vs. PRO + SCH23390 + DA: 88.37 ± 21.13 pA; *n* = 9, *p* = 0.036; Figure 4M). However, no significant changes were observed in the decay-time (SCH23390 + DA: 12.16 ± 3.89 ms vs. PRO + SCH23390 + DA: 10.31 ± 4.32 ms; *n* = 9, *p* = 0.182; Figure 4N). Moreover, in the presence of SCH23390 and DA, PRO caused a rightward shift in the cumulative distributions of sIPSCs frequency and amplitude, indicating a reduction in sIPSCs frequency accompanied by an increase in amplitude (Figures 4I,J). The cumulative distribution of decay-time for sIPSCs showed minimal variation (Figure 4K).

These results suggest that D1 receptors play a significant role in modulating the effects of PRO on NA (−) neurons in the VLPO, particularly in the presence of DA.

### 3.5 Effects of sulpiride and DA on PRO-induced changes in sEPSCs and sIPSCs in NA (−) neurons

**Figure 5:**
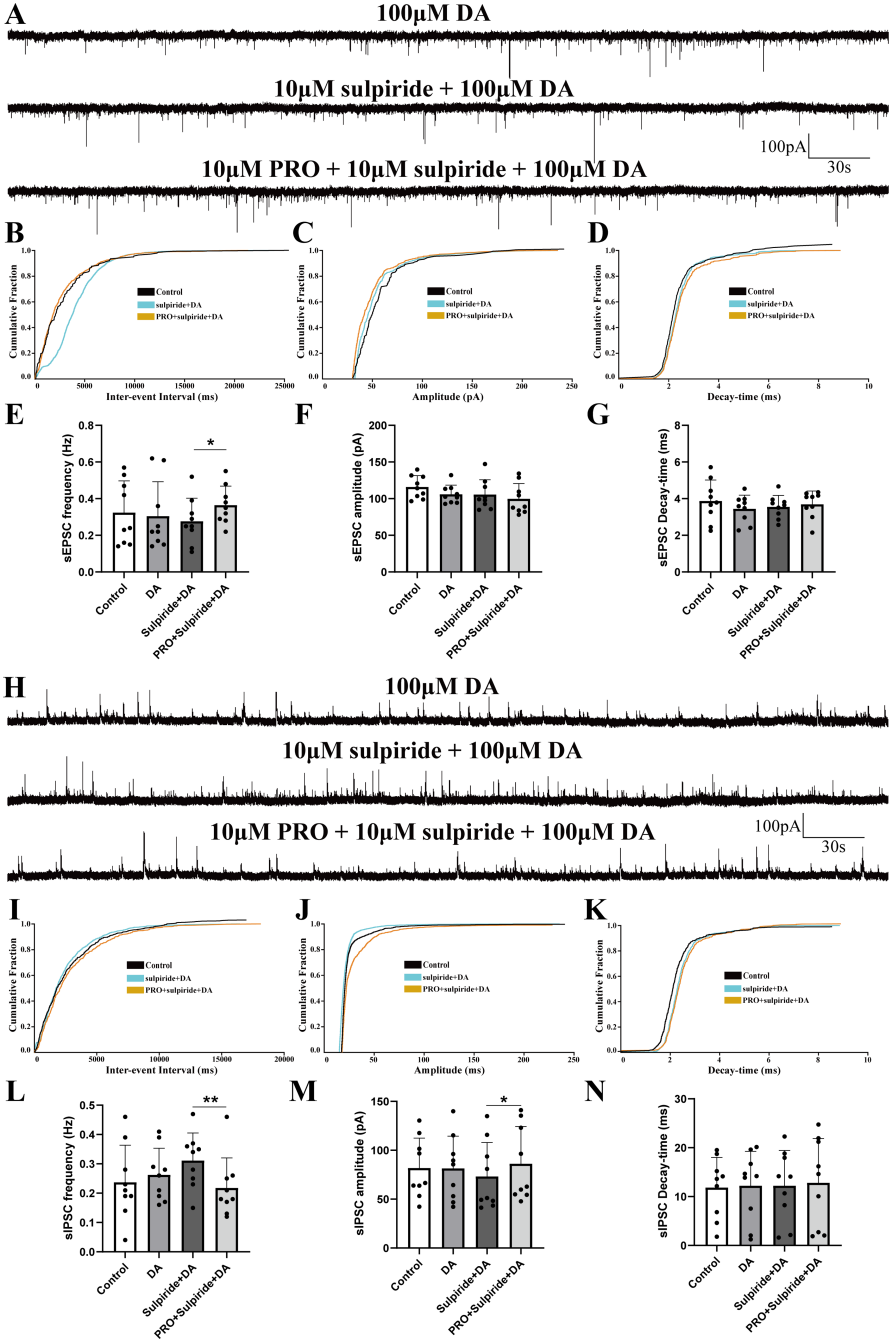
Effects of D2 receptor antagonist sulpiride on PRO-induced changes in sEPSCs and sIPSCs in NA (−) neurons. **(A)** Representative traces showing sEPSCs recorded in NA (−) neurons during sulpiride (10 μM) + DA (100 μM) and PRO (10 μM) + sulpiride + DA application. **(B)** Cumulative probability plot showing the inter-event intervals of sEPSCs during control, sulpiride + DA and PRO + sulpiride + DA conditions. **(C)** Cumulative probability plot of sEPSCs amplitude during control, sulpiride + DA and PRO + sulpiride + DA conditions. **(D)** Cumulative probability plot of sEPSCs decay-time during control, sulpiride + DA and PRO + sulpiride + DA conditions. **(E)** Statistical analysis of sEPSCs frequency before and after PRO + sulpiride + DA application (*n* = 6, ^*^p < 0.05). **(F)** Statistical analysis of sEPSCs amplitude before and after PRO + sulpiride + DA application (*n* = 6, *p* > 0.05). **(G)** Statistical analysis of sEPSCs decay-time before and after PRO + sulpiride + DA application (*n* = 6, *p* > 0.05). **(H)** Representative traces showing sIPSCs recorded in NA (−) neurons during sulpiride (10 μM) + DA (100 μM) and PRO (10 μM) + sulpiride + DA application. **(I)** Cumulative probability plot showing the inter-event intervals of sIPSCs during control, sulpiride + DA and PRO + sulpiride + DA conditions. **(J)** Cumulative probability plot of sIPSCs amplitude during control, sulpiride + DA and PRO + sulpiride + DA conditions. **(K)** Cumulative probability plot of sIPSCs decay-time during control, sulpiride + DA and PRO + sulpiride + DA conditions. **(L)** Statistical analysis of sIPSCs frequency before and after PRO + sulpiride + DA application (*n* = 9, ^**^p < 0.01). **(M)** Statistical analysis of sIPSCs amplitude before and after PRO + sulpiride + DA application (*n* = 9, ^*^p < 0.05). **(N)** Statistical analysis of sIPSCs decay-time before and after PRO + sulpiride + DA application (*n* = 9, *p* > 0.05). Data are presented as mean ± standard deviation and analyzed using one-way ANOVA. ^*^p < 0.05 and ^**^p < 0.01.

In a similar experimental setup, coronal VLPO slices were continuously perfused with ACSF containing the D2 receptor antagonist sulpiride (10 μM) along with DA (100 μM). We observed that the PRO-induced increase in sEPSCs frequency rose from 0.28 ± 0.13 Hz in the sulpiride + DA group to 0.36 ± 0.1 Hz in the PRO + sulpiride + DA group (*n* = 6, *p* = 0.01; Figures 5A,E). However, there were no statistically significant changes in amplitude (sulpiride + DA: 105.51 ± 20.22 pA vs. PRO + sulpiride + DA: 99.76 ± 20.74 pA; *n* = 6, *p* = 0.407; Figure 5F) or decay-time (sulpiride + DA: 3.55 ± 0.62 ms vs. PRO + sulpiride + DA: 3.69 ± 0.73 ms; *n* = 6, *p* = 0.4; Figure 5G). The cumulative probability plot of sEPSCs frequency showed a leftward shift in the presence of PRO + sulpiride + DA, indicating an increase in sEPSCs frequency, with no substantive effect on amplitude or decay-time (Figures 5B–D).

Additionally, PRO reduced the frequency of sIPSCs from 0.31 ± 0.094 Hz in the sulpiride + DA group to 0.22 ± 0.10 Hz in the PRO + sulpiride + DA group (*n* = 9, *p* = 0.003; Figures 5H,L), while the amplitude increased (sulpiride + DA: 73.21 ± 34.79 pA vs. PRO + sulpiride + DA: 86.19 ± 38.07 pA; *n* = 9, *p* = 0.013; Figure 5M). No significant changes were noted in the decay-time (sulpiride + DA: 12.21 ± 7.22 ms vs. PRO + sulpiride + DA: 12.82 ± 9.05 ms; *n* = 9, *p* = 0.764; Figure 5N). In the presence of sulpiride + DA, PRO induced a rightward shift in the cumulative probability of sIPSCs frequency and amplitude, indicating a decrease in sIPSCs frequency and an increase in amplitude, with minimal differences in decay-time (Figures 5I–K).

These results suggest that while D2 receptors are involved in modulating the effects of PRO on NA (−) neurons, their impact is relatively modest, primarily affecting the frequency of sEPSCs and the amplitude of sIPSCs.

### 3.6 The effects of DA and dopaminergic receptors on PRO-induced changes in sEPSCs and sIPSCs of NA (−) neurons

To systematically analyze the role of dopaminergic receptors in regulating the effects of DA on PRO-induced alterations in NA (−) neuronal activity, we compared the potentiation of sEPSCs frequency across different experimental groups. Potentiation was defined as the percentage increase in sEPSCs or sIPSCs frequency compared to the control level (ACSF perfusion).

**Figure 6:**
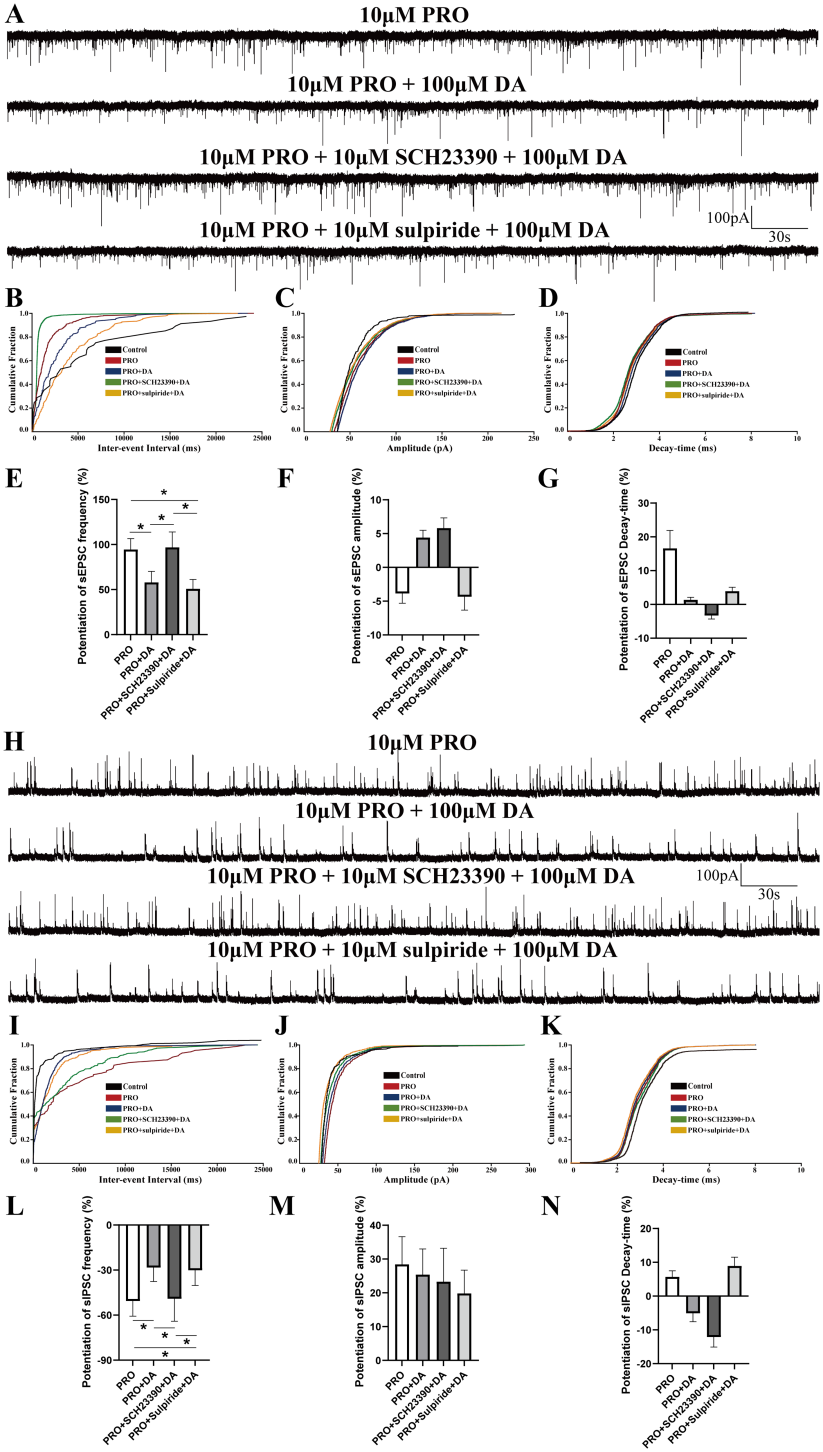
Comparative analysis of DA and dopaminergic receptors’ effects on PRO-induced sEPSCs and sIPSCs in NA (−) neurons. **(A)** Summary of sEPSCs frequency potentiation across different experimental groups. **(B)** Cumulative probability plot of sEPSCs frequency in the PRO, PRO + DA, PRO + SCH23390 + DA, and PRO + sulpiride + DA groups. **(C)** Cumulative probability plot of sEPSCs amplitude across the different groups. **(D)** Cumulative probability plot of sEPSCs decay-time across the different groups. **(E)** Statistical comparison of sEPSCs frequency potentiation among the different groups (*n* = 6, ^*^p < 0.05). **(F)** Statistical comparison of sEPSCs amplitude potentiation among the different groups (*n* = 6, *p* > 0.05). **(G)** Statistical comparison of sEPSCs decay-time potentiation among the different groups (*n* = 6, *p* > 0.05). **(H)** Summary of sIPSCs frequency inhibition ratios across different experimental groups. **(I)** Cumulative probability plot of sIPSCs frequency in the PRO, PRO + DA, PRO + SCH23390 + DA, and PRO + sulpiride + DA groups. **(J)** Cumulative probability plot of sIPSCs amplitude across the different groups. **(K)** Cumulative probability plot of sIPSCs decay-time across the different groups. **(L)** Statistical comparison of sIPSCs frequency inhibition among the different groups (*n* = 6, ^*^p < 0.05). **(M)** Statistical comparison of sIPSCs amplitude potentiation among the different groups (*n* = 6, *p* > 0.05). **(N)** Statistical comparison of sIPSCs decay-time potentiation among the different groups (*n* = 6, *p* > 0.05). Data are presented as mean ± standard deviation and analyzed using one-way ANOVA. ^*^p < 0.05.

As shown in Figures 6A,E, the potentiation of sEPSCs frequency was significantly lower in the PRO + DA group (PRO: 94.38 ± 12.23% vs. PRO + DA: 57.89 ± 12.3%) and the PRO + sulpiride + DA group (PRO: 94.38 ± 12.23% vs. PRO + sulpiride + DA: 50.87 ± 10.32%) compared to the PRO group alone (*n* = 6, *p* < 0.05). However, no significant difference was observed in the PRO + SCH23390 + DA group (PRO: 94.38 ± 12.23% vs. PRO + SCH23390 + DA: 96.77 ± 17.12%; *n* = 6, *p* > 0.05). Additionally, when comparing the PRO + DA group with the PRO + SCH23390 + DA group, the increase in sEPSCs frequency was significantly higher in the latter (PRO + DA: 57.89 ± 12.3% vs. PRO + SCH23390 + DA: 96.77 ± 17.12%; *n* = 6, *p* < 0.05; Figures 6A,E), while no significant difference was found between the PRO + sulpiride + DA group and the PRO + DA group (PRO + DA: 57.89 ± 12.3% vs. PRO + sulpiride + DA: 50.87 ± 10.32%; *n* = 6, *p* > 0.05; Figure 6E). Consistently, the potentiation of sEPSCs frequency was lower in the PRO + sulpiride + DA group compared to the PRO + SCH23390 + DA group (PRO + SCH23390 + DA: 96.77 ± 17.12% vs. PRO + sulpiride + DA: 50.87 ± 10.32%; *n* = 6, *p* < 0.05; Figure 6E).

No statistically significant differences were observed in the potentiation of sEPSCs amplitude or decay-time among the four groups (data not shown; *n* = 6, *p* > 0.05; Figures 6F,G). The cumulative probability plots of sEPSCs were comparable across all groups (Figures 6B–D).

As shown in Figures 6H,L, compared with PRO group, the attenuation of sIPSCs frequency was lower in PRO + DA group (PRO: −50.59 ± 10.12% vs. PRO+DA: −28.34 ± 9.3%; *n* = 6; *p* < 0.05) and PRO + sulpiride + DA group (PRO: −50.59 ± 10.12% vs. PRO + sulpiride + DA: −30.12 ± 10.1%; *n* = 6; *p* < 0.05), whereas no significant effect was detected in PRO + SCH23390 + DA group (PRO: −50.59 ± 10.12% vs. PRO + SCH23390 + DA: −49.10 ± 15.01%; *n* = 6; *p* > 0.05). Meanwhile, compared with PRO + DA group, the suppression ratio of sIPSCs frequency was higher in the PRO + SCH23390 + DA group (PRO + DA: −28.34 ± 9.3% vs. PRO + SCH23390 + DA: −49.10 ± 15.01%; *n* = 6; *p* < 0.05; Figures 6H,L), yet we found slight changes in PRO + sulpiride + DA group (PRO + DA: −28.34 ± 9.3% vs. PRO + SCH23390 + DA: −30.12 ± 10.1%; *n* = 6; *p* > 0.05; Figures 6H,L). Surprisingly, experimental results demonstrated that there were no significant differences in the amplitude and Decay-time increase rate of sIPSCs among the four groups (data not shown; *n* = 6; *p* > 0.05; Figures 6M,N). Cumulative probability plots of sIPSCs were comparable among groups (Figures 6I–K).

Taken together, these results suggest that DA attenuates the PRO-induced facilitation of sEPSCs frequency primarily through D1 receptors rather than D2 receptors.

## 4 Discussion

In this study, we employed *in vitro* patch-clamp techniques to investigate the activation effects of PRO on NA (−) neurons in the VLPO, particularly focusing on the involvement of dopaminergic receptors. We identified two subtypes of VLPO neurons—NA (−) and NA (+)—by recording their ongoing discharges in response to bath-applied NA. Our findings demonstrated that PRO significantly increased the firing rate of NA (−) neurons while reducing the firing rate of NA (+) neurons within the VLPO (Figure 1). The data suggest that PRO activates GABAergic NA (−) neurons by positively modulating GABA_A receptors, leading to a significant increase in sEPSCs frequency and promoting the release of presynaptic glutamate (Figures 2A,E). Furthermore, PRO reduced the release of presynaptic GABA from NA (−) neurons by decreasing sIPSCs frequency while enhancing GABA_A receptor responsiveness, as evidenced by the increased sIPSCs amplitude (Figures 2H,L,M). These results imply that the regulation of PRO’s effects on NA (−) neurons is likely mediated by D1 receptors on these neurons (Figures 4E,L,M). Additionally, the potentiation of sEPSCs frequency and the suppression of sIPSCs frequency induced by PRO were modulated by DA, without affecting the increase in sIPSCs amplitude (Figures 6E,L,M). Notably, these DA effects were abolished by blocking D1 receptors but not D2 receptors. To better illustrate the effects of PRO and DA on VLPO synaptic activity, cumulative fraction plots have been updated to include control (ACSF) values. This addition provides a baseline reference, allowing for a more accurate comparison of the modulation of synaptic properties under different conditions. Thus, our results indicate that PRO excites sleep-promoting NA (−) neurons in the VLPO, at least in part, through D1 receptor-dependent mechanisms.

Our findings demonstrate that DA modulates VLPO neuronal activity, likely mediated by D1 receptors. The VLPO’s reciprocal connections with the VTA and periaqueductal gray suggest a broader regulatory network integrating sleep and arousal states. Previous studies, such as Cornil et al. (2002) and Gordon-Fennell et al. (2020), have demonstrated dopaminergic projections from the VTA to the preoptic area, further supporting the role of DA in modulating VLPO neuronal activity. This evidence highlights the importance of dopaminergic signaling in coordinating sleep-wake transitions and anesthesia.

Understanding the mechanisms underlying general anesthesia remains a critical scientific challenge in both anesthesia and neuroscience (Mashour, 2024). The “shared circuits hypothesis, “which proposes that general anesthesia-induced reversible unconsciousness may be regulated by wake-sleep circuitry, is supported by numerous rodent studies (Bao et al., 2023; Eikermann et al., 2020; Yue et al., 2021; Zhu et al., 2023). Among the various nuclei involved in sleep-arousal regulation, the VLPO stands out as a key hub for modulating both sleep and anesthesia (Liang et al., 2021; Prokofeva et al., 2023; Luo et al., 2023). Given that sleep-active GABAergic neurons in the VLPO have reciprocal inhibitory connections with wake-active dopaminergic neurons in the VTA and vPAG (Bao et al., 2023; Yang et al., 2022; Weber and Dan, 2016). The significance of DA signaling in the VLPO lies in its ability to modulate synaptic activity in sleep-promoting GABAergic neurons, which are central to sleep-wake regulation (Bao et al., 2023; Yang et al., 2022). This modulation is facilitated by dopaminergic projections from upstream nuclei, such as the VTA and vPAG (Yang et al., 2022; Weber and Dan, 2016), which release DA onto VLPO neurons. These dopaminergic inputs are part of a broader regulatory network that integrates arousal and sleep-promoting circuits. Therefore, understanding DA’s role in the VLPO provides critical insights into the mechanisms underlying the shared circuits of sleep and anesthesia. While our findings provide pharmacological evidence of DA’s impact on VLPO neuronal activity, the direct source of DA release in the VLPO warrants further investigation. Upstream dopaminergic neurons in the VTA and vPAG are likely contributors, given their anatomical projections to the VLPO and their role in sleep-wake regulation. To confirm this, future experiments could utilize optogenetic or chemogenetic stimulation of dopaminergic neurons in these upstream nuclei while monitoring DA release using *in vivo* microdialysis or amperometry (Song et al., 2024). Concurrent electrophysiological recordings in the VLPO could further elucidate the functional impact of DA signaling on VLPO neurons. To validate the role of DA signaling in modulating VLPO synaptic activity, future studies should consider incorporating optogenetic or chemogenetic activation of upstream dopaminergic neurons, such as those in the VTA or vPAG. Simultaneous electrophysiological recordings in the VLPO would allow for direct assessment of DA’s effects on neuronal activity. Pharmacological blockade of specific DA receptor subtypes during these experiments would help determine the receptor-specific contributions to synaptic modulation. Additionally, molecular techniques, including single-cell RNA sequencing or immunohistochemistry, could confirm the presence of dopaminergic receptors in VLPO neurons, further strengthening the understanding of DA’s significance in this circuit (Chung et al., 2017). We hypothesized that PRO-induced general anesthesia might be associated with dopamine receptors expressed on VLPO neurons.

To test this hypothesis, we classified VLPO neurons into NA (−) and NA (+) subtypes based on their morphological, pharmacological, and electrophysiological properties (Figures 1A,B,E,F). Our data suggest that GABAergic NA (−) neurons in the VLPO play a crucial role in mediating anesthesia, consistent with our earlier findings (Liu et al., 2017). The VLPO has traditionally been divided into different neuronal subpopulations, including GABAergic, glutamatergic, and galaninergic neurons (Kostin et al., 2023; Chung et al., 2017; Oishi et al., 2023; Moffitt et al., 2018). Our results indicate that two-thirds of VLPO GABAergic NA (−) neurons, which are multipolar or triangular with low-threshold spikes (LTS), are inhibited by NA under current-clamp conditions (Figures 1A,B). Additionally, 5-HT increases sIPSCs frequency in putative sleep-promoting (PSP) VLPO-LTS cells, while general anesthesia-activated NA (−) neurons can be categorized into 5-HT-inhibited type-1 or 5-HT-excited type-2 PSP cells (Fan et al., 2023; Weber and Dan, 2016). Recent advancements in optogenetic or chemogenetic manipulations and single-cell RNA-sequencing have revealed significant heterogeneity within the VLPO, highlighting the presence of both inhibitory and excitatory neurons with distinct neuroregulatory characteristics (Chung et al., 2017; Moffitt et al., 2018). Accumulating evidence suggests that GABAergic NA (−) neurons are also implicated in anesthesia, consistent with our observations (Figure 2).

However, recent studies challenge the notion that anesthesia and sleep share the same neural circuits. Some researchers have proposed that anesthesia and sleep might engage different regulatory pathways in VLPO neurons (Vanini et al., 2020). This divergence highlights the complexity of the VLPO’s role in sleep and anesthesia and underscores the need for further investigation.

In line with our previous whole-cell patch-clamp research (Liu et al., 2017), we observed that PRO robustly activated NA (−) neurons while inhibiting NA (+) neurons (Figures 1C,D,G,H). This was reflected in the increased spontaneous firing rate and membrane depolarization of NA (−) neurons, contrasted with the reduced firing rate and membrane hyperpolarization of NA (+) neurons (Figures 1C,G). These findings suggest that PRO inhibits NA (+) neurons, potentially leading to the disinhibition and activation of NA (−) neurons. This disinhibition mechanism may underlie the sleep-anesthesia relationship in the VLPO.

Contrary to our findings, Vanini et al. (2020) reported that activation of GABAergic or glutamatergic neurons in the VLPO and MnPO influenced sleep-wake architecture but did not impact isoflurane anesthesia transitions. To reconcile these discrepancies, we measured the changes in frequency, amplitude, and decay-time of sEPSCs and sIPSCs in VLPO NA (−) neurons following PRO and/or other chemical applications in whole-cell current-clamp mode (Figure 2). Our electrophysiological analyses indicated that sEPSCs and sIPSCs are primarily mediated by AMPA receptors and GABA_A receptors, respectively (Figures 2A,H). PRO significantly increased sEPSCs frequency in NA (−) neurons, without altering amplitude or decay-time, suggesting enhanced glutamatergic transmission (Figures 2E–G). Conversely, PRO decreased sIPSCs frequency while increasing amplitude, without affecting decay-time, indicating that PRO modulates GABAergic transmission in these neurons (Figures 2L–N).

The role of GABA_A receptors in mediating PRO’s effects was further confirmed by the complete abolition of sEPSCs when GABAzine was applied, supporting the notion that PRO induces LOC through positive modulation of GABA_A receptors (Kim et al., 2020). Previous studies have demonstrated that microinjection of a GABA_A agonist into the VLPO accelerates PRO-mediated loss of righting reflex (LORR) and delays recovery, while GABA_A antagonists produce the opposite effects (Yuan et al., 2017). This highlights the central role of GABA_A receptors in mediating PRO’s anesthetic effects.

Our study also explored the involvement of dopaminergic receptors in PRO-induced activation of VLPO neurons. Recent tract-tracing studies have shown that sleep-promoting VLPO GABAergic neurons are anatomically connected to wake-active dopaminergic neurons in the VTA and vPAG (Yang et al., 2022; Weber and Dan, 2016), suggesting the expression of dopamine receptors in VLPO neurons. Our findings indicate that DA, acting through D1 receptors, partially offsets the PRO-induced potentiation of sEPSCs frequency and suppression of sIPSCs frequency in NA (−) neurons, without affecting amplitude or decay-time (Figures 3–6). The persistence of PRO-induced changes in sEPSC frequency despite D1 receptor antagonism suggests that other GPCR-mediated pathways may contribute to these effects (Figure 4). Potential candidates include D5 dopaminergic receptors or adrenergic β1/β2 receptors (Tocut et al., 2022), both of which are expressed in sleep-wake regulatory circuits. Additionally, serotonergic receptors may play a modulatory role, given their established influence on VLPO neuronal activity (Sangare et al., 2016). Future investigations using genetic knockouts, selective receptor antagonists, or chemogenetic approaches are necessary to delineate the contributions of these non-D1 receptor pathways to PRO-induced modulation of VLPO neurons. Our findings reveal that D1 receptor inhibition reverses DA-induced suppression of sEPSC frequency, even in the absence of PRO, indicating a baseline regulatory role for D1 receptors in VLPO neuronal activity (Figure 6). This suggests that D1 receptors not only modulate anesthetic-induced changes but also play a fundamental role in maintaining the basal synaptic dynamics of NA (−) neurons. The interplay between baseline dopaminergic signaling and PRO-induced modulation highlights the complexity of these circuits and underscores the need for further investigation into how these mechanisms integrate to regulate sleep and anesthesia. To strengthen the conclusion regarding the specificity of D1 receptor-mediated mechanisms, future studies should incorporate advanced methodologies such as optogenetics, chemogenetics, or D1 receptor knockout models. These approaches would allow for precise dissection of D1 receptor contributions to both baseline and PRO-modulated neuronal activity, providing deeper insights into the role of dopaminergic signaling in VLPO circuits during anesthesia. These results suggest that DA modulates the excitatory effects of PRO on NA (−) neurons, likely through a D1 receptor-dependent mechanism.

Our previous work (Zhang et al., 2020; Zhang et al., 2021), combining *in vivo* fiber photometry, EEG recordings, microinjection of D1 receptor modulators into the nucleus accumbens (NAc), and *ex vivo* brain slice techniques, demonstrated that NAc GABAergic neurons expressing D1 receptors play a role in regulating emergence from PRO and isoflurane anesthesia. Similar findings have been reported in studies examining the role of GABA_A receptors in regulating arousal and anesthetic state transitions (Liu et al., 2020). However, the precise mechanisms by which DA modulates PRO’s effects on VLPO neurons remain to be fully elucidated.

Our study has some limitations. Although we employed comprehensive methods to identify sleep-promoting GABAergic neurons in the VLPO, technical limitations may have affected our ability to precisely delineate neuronal subpopulations. Future studies should utilize advanced techniques, such as optogenetics, chemogenetics, and transgenic models, to further refine the understanding of these neurons’ roles in anesthesia. Additionally, direct evidence of reciprocal projections between the VLPO and dopaminergic nuclei like the VTA and vPAG is still lacking. Advanced transsynaptic circuit tracing, coupled with *in vivo* and *ex vivo* electrophysiological recordings, could help address these gaps and provide a more detailed understanding of the neural mechanisms underlying general anesthesia.

## 5 Conclusion

In summary, our study provides compelling evidence that PRO activates NA (−) neurons and inhibits NA (+) neurons in the VLPO through GABA_A receptors. The excitatory effects of PRO on sleep-promoting NA (−) neurons appear to be modulated by D1 receptors, highlighting the potential role of these receptors in regulating anesthesia-induced activation of VLPO neurons. These findings contribute to a deeper understanding of the neural mechanisms underlying general anesthesia and suggest potential therapeutic targets for managing anesthesia-related disorders of consciousness.

## Data Availability

The original contributions presented in the study are included in the article/supplementary material, further inquiries can be directed to the corresponding author.
